# Computed Tomography-Based Radiomics Diagnostic Model for Fat-Poor Small Renal Tumor Subtypes

**DOI:** 10.3390/diagnostics15111365

**Published:** 2025-05-28

**Authors:** Seokhwan Bang, Heehwan Wang, Hoyoung Bae, Sung-Hoo Hong, Jiook Cha, Moon Hyung Choi

**Affiliations:** 1 Department of Urology, Seoul St. Mary’s Hospital, College of Medicine, The Catholic University of Korea, Seoul 06591, Republic of Korea; drvion@gmail.com (S.B.); toomey@catholic.ac.kr (S.-H.H.); 2 Interdisciplinary Program in Artificial Intelligence, Seoul National University, Seoul 08826, Republic of Korea; dhkdgmlghks@snu.ac.kr; 3 Department of Urology, Seoul Metropolitan Government, Seoul National University Boramae Medical Center, Seoul 07061, Republic of Korea; hoyoung.b@gmail.com; 4 Department of Phychology, Seoul National University, Seoul 08826, Republic of Korea; 5 Department of Radiology, Eunpyung St. Mary’s Hospital, College of Medicine, The Catholic University of Korea, Seoul 06591, Republic of Korea

**Keywords:** RCC, small renal mass, radiomics, kidney cancer, diagnostics

## Abstract

**Background:** Differentiating histologic subtypes of fat-poor small renal masses using conventional imaging remains difficult due to their overlapping radiologic characteristics. We aimed to develop a machine learning-based diagnostic model using CT-derived radiomic features to classify the five most common renal tumor subtypes: clear cell RCC (ccRCC), papillary RCC (pRCC), chromophobe RCC (chRCC), angiomyolipoma (AML), and oncocytoma. **Methods:** A total of 499 patients with pathologically confirmed renal tumors who underwent preoperative contrast-enhanced CT and nephrectomy were retrospectively analyzed. **Results:** We extracted and analyzed radiomic features from 1548 multi-phase CT scans from 499 patients, focusing on fat-poor tumors. Five machine learning classifiers including Linear SVM, Rbf SVM, Random Forest, and XGBoost were involved. Among the models, XGBoost showed the best classification performance, with an average AU-PRC: mean = 0.757, standard error = 0.033 and a renal angiomyolipoma-specific AU-ROC: mean = 0.824, standard error = 0.023. These results outperformed other single-phase CT radiomic feature-based machine learning models trained with 20% of principal components. **Conclusions:** This study demonstrates the effectiveness of radiomics-based machine learning in classifying renal tumor subtypes and highlights the potential of AI in medical imaging. The findings, particularly the utility of single-phase CT and feature optimization, offer valuable insights for future precision medicine approaches. Such methods may support more personalized diagnosis and treatment planning in renal oncology.

## 1. Introduction

Kidney cancer represents a significant global health burden, with the American Cancer Society reporting an estimated 81,610 new cases diagnosed in the United States alone in 2024. This highlights the urgent need for advanced diagnostic and treatment strategies to improve patient outcomes. Kidney cancer, predominantly renal cell carcinoma (RCC), is complex due to its multiple subtypes, each with unique prognostic implications [1,2].

However, considered anatomically, the kidney is surrounded by a membrane called Gerota’s fascia, and this membrane plays a role in preventing the spread of kidney cancer, so inserting a needle to perform a biopsy is taboo. For this reason, imaging diagnosis is very important in kidney cancer. Kidney cancer is usually discovered by ultrasound sonography or CT scan accidentally, and when a renal mass is diagnosed as cancer, the accuracy is currently known to be about 90% [3]. However, this high diagnosis rate includes all cases that can be easily diagnosed as cancer, such as very large kidney tumors. In cases such as small kidney tumors or fat-poor tumors, the diagnosis rate drops sharply [4]. When pathological results cannot be predicted, partial nephrectomy or radical nephrectomy, which are chosen for proactive surgical management, are highly invasive procedures that require advanced surgical expertise. If the lesion turns out to be benign, these surgeries can impose significant health and economic burdens on the patient. Therefore, accurate screening through imaging studies is of paramount importance.

Accurate diagnosis of kidney cancer subtypes remains challenging due to the variability in their appearance on imaging modalities like computed tomography (CT). This variability can lead to differential diagnostic outcomes, affecting treatment decisions [5,6]. Studies such as those by Bauman et al. [7] and Tanaka et al. [8] have documented the incidence and challenges of accurately classifying these tumors, emphasizing the risk of unnecessary interventions due to diagnostic inaccuracies.

The integration of machine learning with radiomic data represents a cutting-edge approach in the diagnosis of kidney cancer. Deep learning models, particularly those employing convolutional neural networks (CNNs), have demonstrated superior accuracy in identifying and classifying renal tumors compared to traditional methods [9,10]. Such models not only streamline the diagnostic process, but also reduce the variability associated with human interpretation [11,12].

Our research team has also made various attempts to utilize AI in interpreting CT scans to classify the presence of renal tumors and their subtypes. Our team first attempted prediction using Convolutional Neural Networks (CNN), one of the AI techniques known for achieving the highest predictive accuracy. Although we achieved an accuracy of nearly 62%, we were unable to obtain actionable feedback from this approach. However, this study confirmed that predictions are feasible, and that the predictive accuracy of the AI model exceeded that of current radiologists [13]. This model addresses critical gaps in current diagnostic approaches by providing a robust, automated system that offers high accuracy and reproducibility [14]. However, this study concluded that it could not provide feedback substantial enough to advance current medical practice. While the CNN demonstrated good predictive performance, it lacked the capability to offer actionable feedback or highlight specific components for radiologists to focus on.

Radiomics has emerged as a revolutionary technique in medical imaging, offering the potential to extract quantifiable data from images that are beyond human visual detection. This approach has significantly advanced the classification of renal masses, aiding in distinguishing between benign and malignant forms [15]. Sun et al. [16] and Leon et al. [17] provide evidence of the effectiveness of radiomic features in enhancing the diagnostic precision of RCC.

Radiomics, an AI-driven technology, offers significant potential as a supportive tool for radiologists. Its diagnostic decisions are based on features that can be validated by human experts, making it a reliable feedback mechanism. Leveraging this technology, we explored its application in diagnosing subtypes of renal cancer. We hypothesized that integrating radiomics into clinical practice could enhance the diagnostic accuracy of radiologists. As a precursor to this study, we first conducted research focused solely on determining the presence or absence of tumors [18]. Through this approach, we aimed to identify, using radiomics, features that should be prioritized for classification. This study is also part of the process of verifying those features.

By improving the accuracy of subtype classification, our model contributes to more tailored treatment strategies, potentially leading to better patient outcomes. The adaptability of our model across different imaging modalities and its application to other cancer types also suggests a broad potential impact, paving the way for future innovations in oncological diagnostics [19,20].

## 2. Materials and Methods

### 2.1. Patients

We utilized data from patients who underwent total or partial renal resection at a single institution between 2003 and 2021. Excluded from the study were cases of kidney tumor with a fat component exceeding 30%. Only patients with fat-poor tumors were included, resulting in a total selection of 499 patients. The average age of these patients was 56.02 ± 12.18 years, and the mean tumor size on CT was 3.515 ± 2.42 cm. The study encompassed patients with benign tumors like oncocytoma and AML, as well as those with malignant tumors such as clear cell, chromophobe, and papillary-type RCC. Each patient underwent CT imaging in one to four phases, resulting in a total of 1548 sets of CT images. Details of the patient demographics are provided in Table 1.

CT scans were obtained in various configurations: non-contrast, arterial phase (20–30 s after contrast injection), portal phase (60–70 s), and delayed phase (>180 s). For each CT scan, voxel-level segmentation labels were collected, with trained annotators manually outlining kidneys and tumors in the images. Annotations were further reviewed and refined by a radiologist with 11 years of experience. A second diagnosis was provided only if the radiologist had significant doubts about the initial diagnosis. Radiologist performance was evaluated based on the first diagnosis (top-one performance) and both the first and second diagnoses (top-two performance).

### 2.2. Radiomics Feature Analysis Workflow

**Figure 1:**
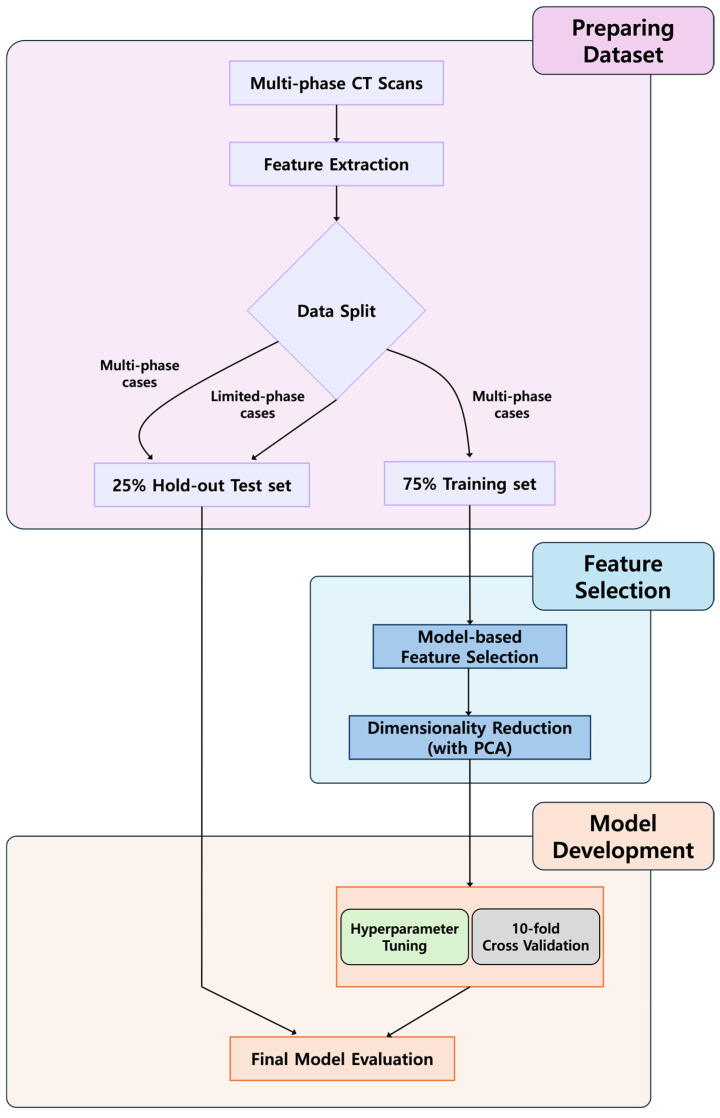
Flow chart of radiomic feature analysis. Radiomics features extracted from multi-phase CT scans were used to develop machine learning models for classifying renal cell tumor subtypes. Participants with multi-phase scans were split into training (75%) and hold-out test (25%) sets, while those with single- or two-phase scans were included only in the test set. To address class imbalance, synthetic oversampling was applied during model training and feature selection.

Radiomics quantitatively extract textural information from medical images, which can then be utilized further by machine learning algorithms to support clinical decision-making. To develop multi-phase CT scan radiomic feature-based machine learning models for classifying subtypes of renal cell tumors, we initially obtained various types of radiomic features from each phase of the CT scans. Considering that some participants only underwent one or two phases of CT scans, such as the non-contrast and portal phases, we split participants with multi-phase and those with limited phase CT scans differently into training and hold-out test sets to align with our objective of developing machine learning models based on multi-phase CT scan radiomic features. We first randomly split participants with multi-phase CT scans into 75% of the training set and 25% of the hold-out test set in a stratified manner, and then we included those with single-phase or two-phase phase CT scans only in the hold-out test set. This resulted in both the training and the hold-out test set having a similar number of participants. The training and hold-out test sets consisted of similar proportions of renal cell tumor subtypes. Only the training set was used for feature selection and model training with 10-fold cross-validation. We compared the following four most popular machine learning algorithms: Linear Support Vector Machine (Linear SVM), Radial basis function Support Vector Machine (Rbf SVM), Random Forest, and XGBoost [21]. We conducted hyperparameter tuning through 50 trials of a random hyperparameter search for renal cell tumor subtype classification. We searched hyperparameters with Optuna (version 3.1.0) [22]. Codes used for our analyses are available for reproducibility testing (https://github.com/Transconnectome/Kidney_Radiomics; accessed on 9 February 2025). Due to the class imbalance among renal cell tumor subtypes, with a great number of participants having specific renal cell tumor subtypes such as clear cell renal cell carcinoma compared to other subtypes, we employed oversampling during the training phase of model-based feature selection and model development. We implemented oversampling with the Synthetic Minority Over-sampling Technique (SMOTE) using imbalanced-learn (version 0.10.1) [23]. We evaluated the classification performance of our machine learning models based on their ability to accurately differentiate each renal cell tumor subtype from the others. To assess this, we calculated the evaluation metrics, including accuracy, f1 score, Area Under Receiving Operating Characteristic Curves, and Area Under the Precision and Recall Curve, using a One-vs-Rest approach and the “micro” options in scikit-learn. The hold-out test set was exclusively utilized to evaluate the final performance of machine learning models. By using scikit-learn (version 1.2.1) [24] and Python (version 3.10.8), we conducted feature selection, machine learning model development, and model evaluation processes. We followed the Checklist for Artificial Intelligence in Medical Imaging (CLAIMS) to ensure the reproducibility of our study [25]. A schematic overview of the radiomics feature analysis workflow is illustrated in Figure 1.

### 2.3. Radiomics Feature Extraction

**Figure 2:**
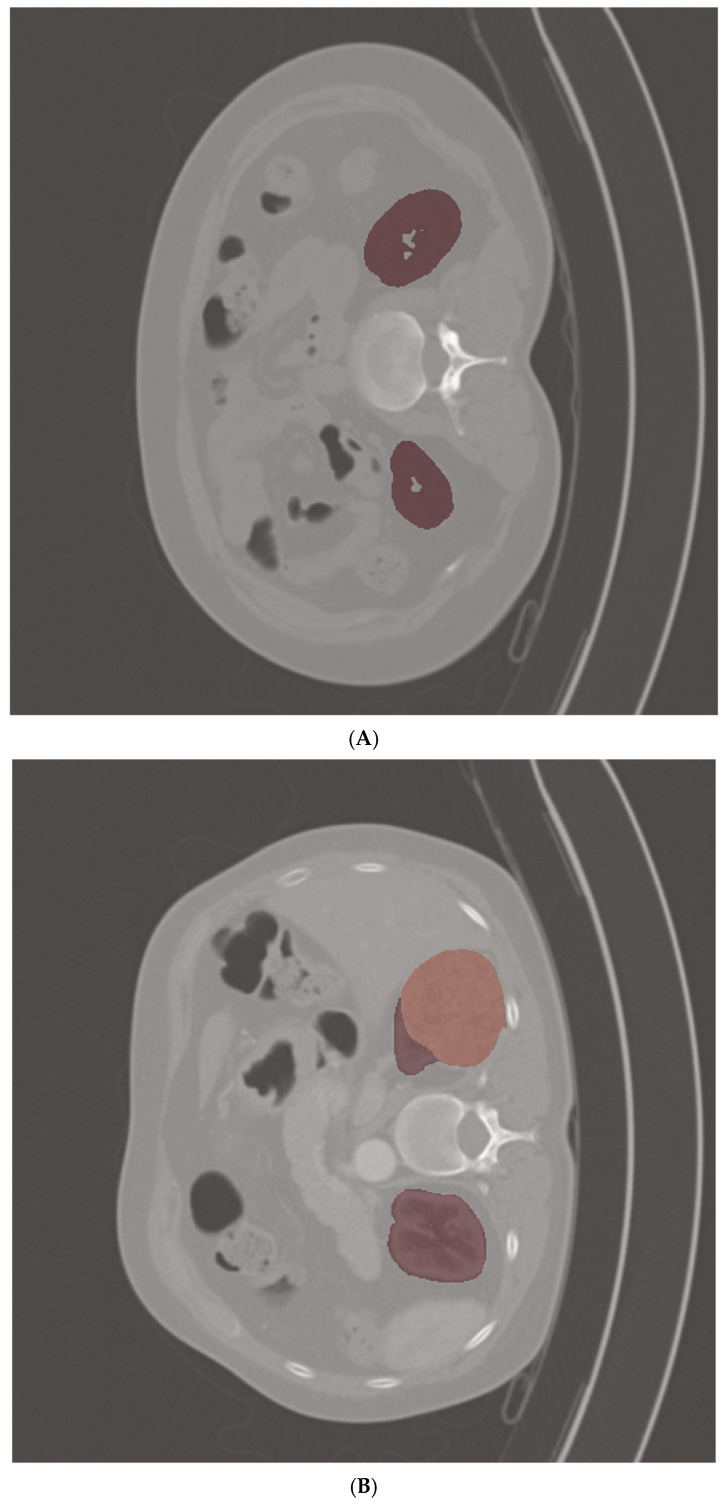

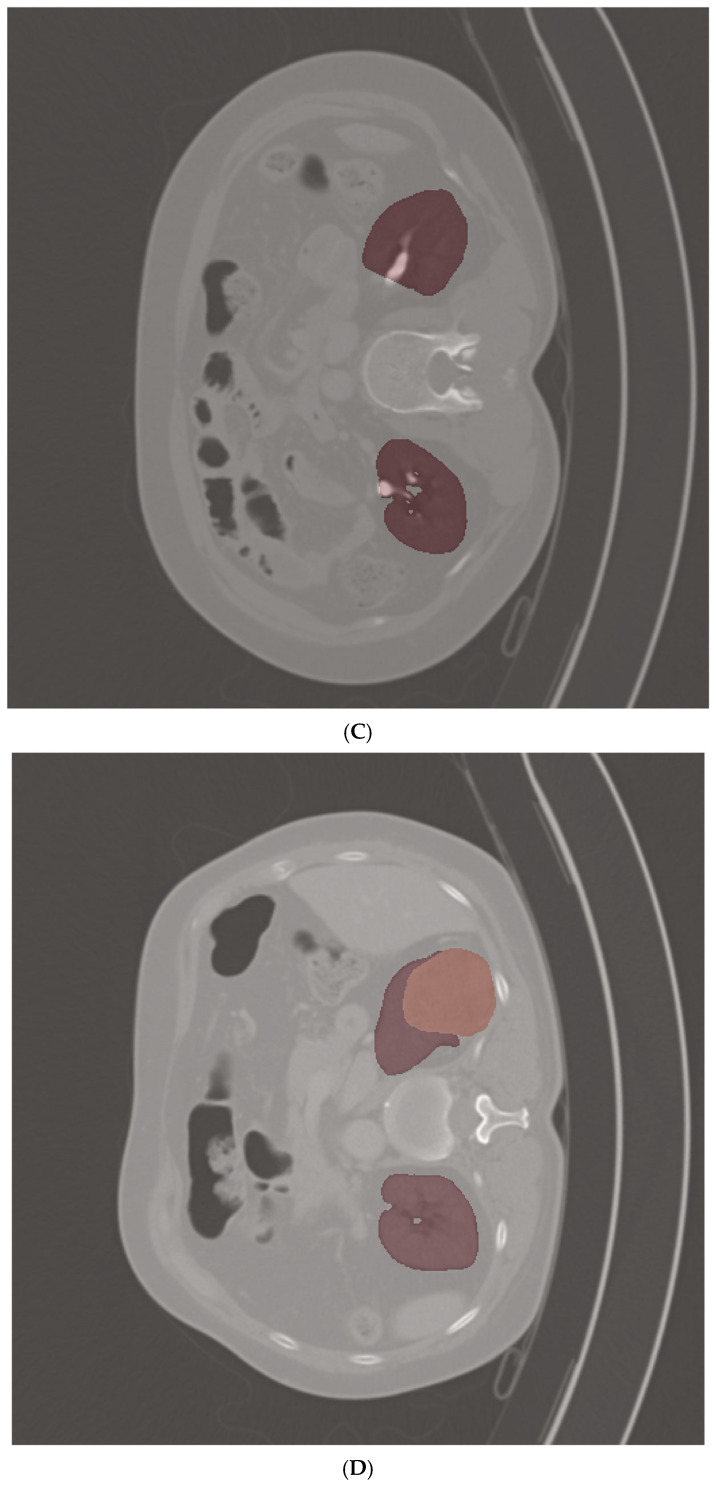
Region of interest (ROI) masks for radiomic feature extraction. (**A**) Non-contrast phase CT scans overlaid with kidney ROI mask. (**B**) Arterial phase CT scans overlaid with kidney ROI mask. (**C**) Delayed phase CT scans overlaid with kidney ROI mask. (**D**) Portal phase CT scans overlaid with kidney ROI mask.

We resampled 1548 multi-phase CT scans with a resolution of 1 mm × 1 mm × 1 mm, and then we extracted 1288 radiomic features from the segmented region of interests (ROIs) of original CT scans, wavelet-filtered CT scans, and Laplacian of Gaussian-filtered scans. We extracted radiomic features utilizing the Python package PyRadiomics (version 3.1.0) [26] and Python (version 3.7). The extracted radiomic features included first-order features, three-dimensional shape features, Gray Level Cooccurence Matrix (GLCM), Gray Level Run Length Matrix (GLRM), Gray Level Size Zone Matrix (GLSZM), Neighboring Gray Tone Difference Matrix (NGTDM), and Gray Level Dependence Matrix (GLDM). Representative multi-phase CT scans with corresponding kidney ROI masks used for radiomic feature extraction are shown in Figure 2.

### 2.4. Feature Selection and Dimensionality Reduction

To ensure that the hold-out test set remained independent and unbiased for evaluating the final model’s performance, we utilized several feature selection methods and a dimensionality reduction method solely on the training set. In the feature selection process, ANOVA F-test with FDR correction using the Benjamin–Hochberg procedure (P_fdr_ = 0.05) was performed on 1288 z-score normalized radiomic features derived from each CT phase. This selected 846, 773, 660, and 972 features from the arterial, delayed, non-contrast, and portal phase scans, respectively. Principal Component Analysis (PCA) was then performed on those selected radiomic features to reduce dimensionality. In this study, we aimed to compare the predictive performance of machine learning models with respect to the number of principal components used, a hyperparameter typically determined by researchers using rule-of-thumb approaches [27,28]. We set the number of principal components to values corresponding to 10% and 20% of the count of features selected via the F-test. When the number of principal components was set to 10% of the selected feature count, the final feature counts were 84, 77, 66, and 97 for arterial, delayed, non-contrast, and portal phase scans, respectively. When set to 20%, the final feature counts were 169, 154, 132, and 194 for the corresponding phases. The PCA results from the training set indicated that setting the number of principal components to 10% and 20% of the count of selected features was appropriate for representing radiomic features selected via the F-test while effectively reducing the dimensionality of the feature space (with the accumulated variance of components were almost 100%) ( Figures S1 and S2). During development of the machine learning models, we assessed the evaluation performance of machine learning models in relation to the number of principal components used. We standardized these principal components (i.e., PCA whitening) for further analyses. Additionally, we employed the Sequential Feature Selector (SFS) on these standardized principal components to identify those that could optimize model evaluation performance on the validation set. The SFS method systematically chose features through a sequential removal process, based on their impact on model performance in the validation set.

### 2.5. Reproducibility and Ethical Considerations

All processes from feature extraction to model evaluation adhered to Checklist for Artificial Intelligence in Medical Imaging (CLAIM) standards to ensure reproducibility and ethical compliance [25]. Our codes are publicly available for validation and further research at GitHub Repository.

## 3. Results

### 3.1. Demographic Characteristics of Patients

In total, 499 patients were included in the development of our multi-phase CT radiomics machine learning model for renal cell tumor subtype classification. Participants with either single-phase or two-phase CT scans were included solely in the hold-out test set. Meanwhile, those with multi-phase CT scans were divided using a stratified approach, with 75% allocated to the training dataset and the remaining 25% reserved for the hold-out test dataset (N_training_ = 289, N_test_ = 210). There were no significant statistical differences in demographic or pathological characteristics between the training and hold-out test datasets (*p* > 0.05) (Table 1).

### 3.2. Multi-Phase CT Radiomic Features for Renal Cell Tumor Subtype Classification

We compared the classification performance on a hold-out test set across various machine learning models with multiple evaluation metrics. The XGBoost model, trained on 20% of principal components from selected radiomic features (XGBoost_20%_), outperformed other algorithms in classifying renal cell tumor subtypes, achieving the highest average accuracy (ACC: mean = 0.475, standard error = 0.011), Area Under Receiving Operating Characteristic Curves (AU-ROC: mean = 0.744, standard error = 0.004), Area Under the Precision and Recall Curve (AU-PRC: mean = 0.474, standard error = 0.006), and F1 score (mean = 0.453, standard error = 0.007). Similarly, XGBoost trained on 10% of principal components from selected radiomic features (XGBoost_10%_) also performed well, achieving the highest performance evaluation metrics across ACC (mean = 0.453, standard error = 0.007), AU-ROC (mean = 0.744, standard error = 0.004), AU-PRC (mean = 0.474, standard error = 0.006), and F1 (mean = 0.453, standard error = 0.007). However, XGBoost_20%_ showed higher overall metrics than XGBoost_10%_. These results are detailed in Table 2.

**Figure 3:**
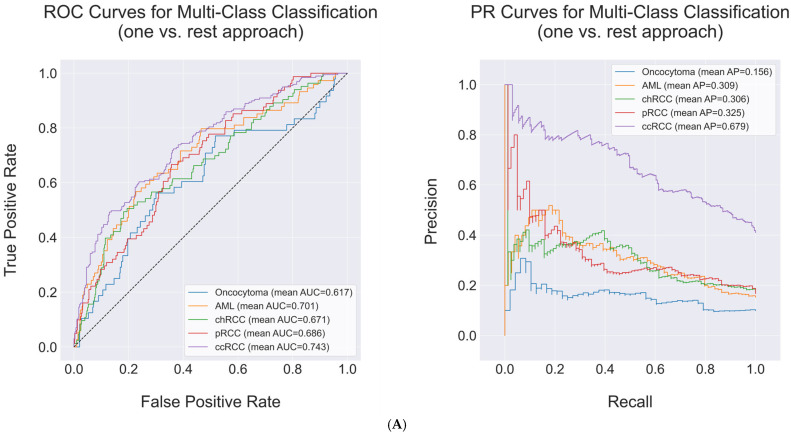

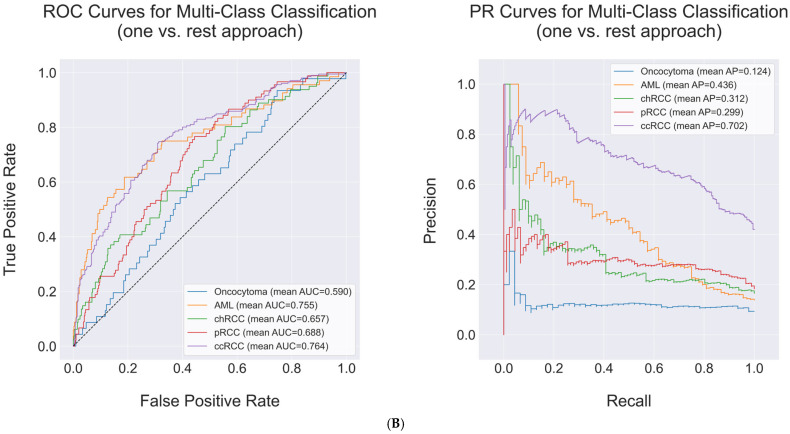
Renal cell tumor subtype classification performance of XGBoost. (**A**) Evaluation performance of machine learning models trained with 10% of the principal components derived from selected radiomic features in stratified 10-fold cross-validation. (**B**) Evaluation performance of machine learning models trained with 20% of the principal components derived from selected radiomic features in stratified 10-fold cross-validation. Abbreviations: AML = renal angiomyolipoma, chRCC = chromophobe renal cell carcinoma, pRCC = papillary renal cell carcinoma, ccRCC = clear cell renal cell carcinoma.

Of note, although the evaluation performance in classifying a specific subtype with other subtypes varied according to the machine learning model and the number of principal components used, XGBoost showed the best performance in classifying clear cell renal cell carcinoma against other subtypes when using either 10% (AU-PRC of XGBoost_10%_: mean = 0.673, standard error = 0.008; AU-ROC of XGBoost_10%_: mean = 0.738, standard error = 0.007) or 20% (AU-PRC of XGBoost_20%_: mean = 0.686, standard error = 0.012; AU-ROC of XGBoost_20%_: mean = 0.750, standard error = 0.011) of principal components for training. For angiomyolipoma (AML), Random Forest (10% principal components) trained with 10% of principal components achieved the highest AU-ROC in classifying AML against other subtypes (mean = 0.769, standard error = 0.009). However, it showed lower AU-PRC in classifying renal angiomyolipoma compared to clear cell renal cell carcinoma when distinguishing these subtypes from others (mean = 0.366, standard error = 0.01). These results indicated that machine learning models were biased towards predicting clear cell renal cell carcinoma, which constitutes the majority of the dataset. The results are detailed in Table 2 and Figure 3.

### 3.3. Single-Phase CT Radiomic Features for Renal Cell Tumor Subtype Classification

In contrast with the results from the multi-phase models, the machine learning models trained on 10% of principal components from selected radiomic features generally outperformed machine learning models trained on 20% of principal components for multiple evaluation metrics. XGBoost, trained on radiomic features derived from arterial and portal phase CT scans, achieved the highest F1 scores (arterial phase: mean = 0.594, standard error = 0.037; portal phase: mean = 0.456, standard error = 0.015) and ACC (arterial phase: mean = 0.594, standard error = 0.037; portal phase: mean = 0.456, standard error = 0.015) compared to other machine learning models trained on arterial and portal phase CT scans radiomic features. Random Forest trained on portal phase CT scan radiomic features showed the highest AU-PRC (portal phase: mean = 0.473, standard error = 0.015) and AU-ROC (portal phase: mean = 0.756, standard error = 0.008). Random Forest trained on radiomic features derived from delayed and non-contrastive phase CT scans also achieved the highest F1 (delayed phase: mean = 0.493, standard error = 0.015; non-contrastive phase: mean = 0.461, standard error = 0.011), AU-PRC (delayed phase: mean = 0.492, standard error = 0.031; non-contrastive phase: mean = 0.471, standard error = 0.013), ACC (delayed phase: mean = 0.493, standard error = 0.015; non-contrastive phase: mean = 0.461, standard error = 0.011), and AU-ROC (delayed phase: mean = 0.766, standard error = 0.016; non-contrastive phase: mean = 0.744, standard error = 0.008).

Similar to models trained on 10% of principal components from selected radiomic features, XGBoost and Random Forest, when trained on 20% of principal components, showed the highest evaluation performance among other machine learning models using the same proportion of principal components. However, their performance was slightly lower compared to models trained with only 10% of principal components. When trained with 20% of principal components derived from arterial phase CT scan radiomic features, XGBoost achieved the best performance across multiple metrics: F1 (arterial phase: mean = 0.531, standard error = 0.002), AU-PRC (arterial phase: mean = 0.565, standard error = 0.014), and ACC (arterial phase: mean = 0.531, standard error = 0.02). XGBoost, when trained with 20% of principal components derived from delayed phase CT scans, achieved the highest AU-ROC (delayed phase: mean = 0.766, standard error = 0.001) compared to other machine learning models trained with the same components. Similarly, XGBoost trained with 20% of principal components derived from portal phase CT scans showed the highest F1 (portal phase: mean = 0.447, standard error = 0.019) and ACC (portal phase: mean = 0.447, standard error = 0.019), while Random Forest achieved the highest AU-PRC (portal phase: mean = 0.449, standard error = 0.014) and AU-ROC (portal phase: mean = 0.735, standard error = 0.007) using the same components. Additionally, Random Forest achieved the highest performance in all metrics when trained with 20% of principal components derived from delayed and non-contrastive phase CT scans: F1 (delayed phase: mean = 0.497, standard error = 0.016; non-contrastive phase: mean = 0.45, standard error = 0.006), AU-PRC (delayed phase: mean = 0.529, standard error = 0.02; non-contrastive phase: mean = 0.433, standard error = 0.009), and ACC (delayed phase: mean = 0.497, standard error = 0.016; non-contrastive phase: mean = 0.45, standard error = 0.006). In the case of AU-ROC, Random Forest, when trained with 20% of principal components derived from either the arterial or the non-contrastive phase, showed the best performance (arterial phase: mean = 0.797, standard error = 0.018; non-contrastive phase: mean = 0.716, standard error = 0.007). The results from single-phase CT radiomic feature-based machine learning models are detailed in Table 3.

In subtype-specific performance, single-phase CT radiomic feature-based machine learning models also performed well on classifying clear cell renal cell carcinoma, similar to the results from multi-phase CT radiomics feature-based machine learning models. Notably, XGBoost, trained with 10% of principal components derived from selected radiomics features extracted from arterial phase CT scans, achieved the highest performance in classifying clear cell renal cell carcinoma (AU-PRC: mean = 0.757, standard error = 0.033) and renal angiomyolipoma (AU-ROC: mean = 0.824, standard error = 0.023). These results outperformed other single-phase CT radiomics feature-based machine learning models trained with 20% of principal components (Table 4A–D; Supplementary Figures S3–S6).

### 3.4. Comparison of Multi-Phase and Single-Phase Models

In summary, performance of machine learning models varied depending on which phase of the CT scan was used for training and which evaluation metric was applied. However, single-phase CT radiomics feature-based models generally outperformed multi-phase models. XGBoost and Random Forest consistently showed better performance than linear SVM and rbf SVM. For single-phase CT scans, machine learning models using 10% principal components derived from selected radiomic features yielded better results. In contrast, for multi-phase scans, models using 20% principal components derived from selected radiomic features performed better. Of note, regardless of the proportion of principal components used, models trained with components derived from single-phase CT scans consistently outperformed those trained with components from multi-phase CT scans. The superior performance of single-phase models, particularly those based on arterial phase scans, suggests that these may be sufficient for accurate renal cell tumor subtype classification, potentially reducing the need for multi-phase CT imaging in clinical practice (Table 4A–D; Supplementary Figures S3–S6).

## 4. Discussion

This study represents a significant step forward in the application of radiomics and machine learning for the classification of renal cell tumor subtypes. By leveraging multi-phase CT scans and advanced machine learning techniques, we have demonstrated a novel approach that has the potential to transform the diagnostic landscape in renal oncology [29,30,31]. Our findings address several critical challenges in the current management of renal tumors. The XGBoost model, trained on 20% of principal components from multi-phase CT radiomic features (XGBoost20%), demonstrated the best overall performance in classifying renal cell tumor subtypes.

Accurate preoperative classification of renal tumor subtypes is a significant clinical challenge, impacting treatment planning and patient outcomes [32,33]. Our radiomics-based approach offers a non-invasive and cross-sectional method to enhance diagnostic accuracy, potentially reducing the need for invasive biopsies and their associated risks [34,35].

The superior performance in identifying clear cell renal cell carcinoma (ccRCC) is particularly noteworthy, given that this subtype is associated with the worst prognosis among RCC subtypes [32]. Early and accurate identification could lead to more aggressive treatment strategies and improved patient outcomes. Our multi-phase CT scans radiomic feature-based machine learning models achieved an AU-PRC of 0.69 and AU-ROC of 0.75 in the independent testing cohort, which represents significant prediction performance.

A key finding of our study is that single-phase CT scans, particularly those from the arterial phase, can achieve comparable or even superior performance to multi-phase models for certain subtypes. The XGBoost model trained on arterial phase CT scans achieved the highest performance in classifying clear cell renal cell carcinoma (AU-PRC = 0.757) and renal angiomyolipoma (AU-ROC = 0.824). This suggests the potential for reduced radiation exposure and shorter imaging protocols, aligning with the broader goals of minimizing patient risk and optimizing resource utilization in healthcare [31,34].

Interestingly, models trained on 10% of principal components generally outperformed those trained on 20% for single-phase CT scans. This suggests that a more parsimonious feature set may suffice for accurate classification, potentially reducing computational complexity and improving model interpretability [33]. This finding opens new avenues for optimizing feature selection in radiomics research.

Our study also highlights the varying levels of model performance across different renal tumor subtypes. While clear cell renal cell carcinoma was consistently well-classified, other subtypes proved more challenging. This pattern aligns with the known heterogeneity of renal tumors and underscores the need for subtype-specific approaches in radiomics research [32,34]. Furthermore, our study adds to the growing body of evidence supporting the utility of radiomics in oncology [30]. By demonstrating that quantitative imaging features can capture clinically relevant information not visible to the naked eye, we reinforce the potential of radiomics to serve as a powerful tool in precision medicine [33].

Despite the promising results, our study has several limitations that warrant discussion. The predominance of ccRCC cases (64.3% in the training cohort) reflects a common challenge in medical imaging studies but may have introduced bias in our models. Our use of single-institution data may limit the generalizability of our findings to different patient populations or imaging protocols. The retrospective nature of our study could have introduced selection bias, and prospective validation studies are necessary to confirm the clinical utility of our approach. We encountered challenges in differentiating among specific malignant subtypes, particularly in identifying oncocytomas (AUC = 0.57–0.69), highlighting the need for further refinement of our models and potentially the incorporation of additional data types. Lastly, while we used an independent hold-out test set, our study lacks external validation on datasets from different institutions or populations, which is crucial for assessing the robustness and generalizability of our findings [33,34].

The challenges we encountered in differentiating among specific malignant subtypes, particularly in identifying oncocytomas (AUC = 0.57–0.69), mirror the difficulties faced in clinical practice. This alignment between computational and human challenges in tumor classification suggests that future improvements in AI models could have direct and significant impacts on clinical decision-making.

Looking ahead, our findings open up several exciting avenues for future research. The exploration of deep learning models, which can learn directly from image data without the need for hand-crafted features, represents a promising next step. Additionally, the integration of radiomics with other data types, such as genomics or clinical information, could lead to more comprehensive and accurate predictive models.

Our study has several significant limitations. First, the predictive accuracy of our research did not reach the commonly accepted threshold of 80%. However, we believe this limitation can be overcome by expanding the dataset, and we are actively collecting additional data to address this issue. Additionally, our study was conducted retrospectively, which means its efficacy has not yet been fully validated. We anticipate that conducting a prospective study based on these data will demonstrate its practical value.

Furthermore, the goal of this study was to identify recommended features using radiomics techniques and utilize feedback to enhance radiologists’ predictive accuracy. However, due to the scope of this research, we were only able to confirm the model’s ability to predict subtypes. We plan to include these broader aspects in future studies to fully realize the potential of this approach.

Ultimately, if we are able to validate our hypothesis step by step, and this predictive model achieves successful outcomes, it will allow us to determine the pathology of renal cancer through imaging studies alone, without the need for biopsy. This advancement would enable us to predict the optimal timing for surgery, prepare appropriate chemotherapy protocols, avoid unnecessary surgeries and their associated risks, and achieve significant economic savings.

## 5. Conclusions

In conclusion, our study not only demonstrates the potential of radiomics-based machine learning in renal tumor classification, but also contributes to the broader dialogue about the role of AI in medical imaging. The patterns we observed, particularly the efficacy of single-phase CT and the potential for feature set optimization, provide valuable insights for future research directions. As we continue to refine these techniques and address current limitations, the promise of more personalized, accurate, and efficient diagnosis and treatment planning in renal oncology comes increasingly within reach. This work represents a crucial step towards realizing the potential of precision medicine in the management of renal tumors, with far-reaching implications for patient care and outcomes.

### Code Availability

The prediction of malignancy in renal tumors by using CT radiomics is found to be feasible. Based on this technology, it is expected that there will be future advances in the diagnosis of renal tumors. The relevant codes are freely available for reproducibility.

## Figures and Tables

**Table 1:** Demographic and pathological characteristics of the patients.

	Training Dataset	Test Dataset	*p* Value
Sex	289	210	0.639
Male (%)	162 (49.2%)	123 (58.6%)	
Female (%)	127 (50.8%)	87 (41.4%)	
Age (years) (range)	57.0 (22, 83)	57.0 (27, 81)	0.816
Cancer size (cm) (range)	3.629 (0.8, 17.5)	3.357 (0.7, 12.0)	0.216
Renal cell tumor subtype			0.881
oncocytoma (%)	25 (8.6%)	23 (11%)	
angiomyolipoma (%)	47 (16.3%)	31 (14.7%)	
chromophobe renal cell carcinoma (%)	48 (16.6%)	36 (17.1%)	
papillary renal cell carcinoma (%)	50 (17.3%)	39 (18.6%)	
clear cell renal cell carcinoma (%)	119 (41.2%)	81 (38.6%)	

We used paired the sample t-test or chi-squared test to hold-out test the differences in demographic and pathological characteristics for continuous variables (i.e., age, cancer size) and categorical variables (i.e., sex, kidney cancer subtype).

**Table 2:** Model performance of multi-phase CT radiomic features-based machine learning models in renal cell tumor subtype classification. We compared the model performance of four different types of machine learning algorithms in classifying the subtype of malignant kidney cancer. The average and standard error over test performance of each model in 10-fold cross-validation on a hold-out test set were calculated. (**A**,**C**) show overall evaluation performance of machine learning algorithms trained with 10% and 20% of principal components, respectively. (**B**,**D**) show the performance of machine learning algorithms in classifying renal cell tumor subtype via a one vs. rest approach, trained with 10% and 20% of principal components, respectively. Abbreviations: AML = renal angiomyolipoma, chRCC = chromophobe renal cell carcinoma, pRCC = papillary renal cell carcinoma, ccRCC = clear cell renal cell carcinoma. Values following signs indicate standard error.

**(A)**										
			**F1**		**AU-PRC**		**ACC**		**AU-ROC**	
Linear SVM			0.423 ± 0.008		0.393 ± 0.016		0.423 ± 0.008		0.703 ± 0.009	
Rbf SVM			0.433 ± 0.017		0.408 ± 0.011		0.433 ± 0.017		0.722 ± 0.006	
XGBoost			0.453 ± 0.007		0.474 ± 0.006		0.453 ± 0.007		0.744 ± 0.004	
Random Forest			0.444 ± 0.009		0.443 ± 0.006		0.444 ± 0.009		0.742 ± 0.003	
**(B)**										
	**AU-PRC**					**AU-ROC**				
	**Oncocytoma**	**AML**	**chRCC**	**pRCC**	**ccRCC**	**Oncocytoma**	**AML**	**chRCC**	**pRCC**	**ccRCC**
	**vs. Rest**	**vs. Rest**	**vs. Rest**	**vs. Rest**	**vs. Rest**	**vs. Rest**	**vs. Rest**	**vs. Rest**	**vs. Rest**	**vs. Rest**
Linear SVM	0.096 ± 0.01	0.285 ± 0.039	0.257 ± 0.044	0.246 ± 0.022	0.613 ± 0.019	0.511 ± 0.042	0.688 ± 0.03	0.605 ± 0.037	0.607 ± 0.023	0.697 ± 0.011
Rbf SVM	0.101 ± 0.014	0.292 ± 0.017	0.242 ± 0.013	0.276 ± 0.02	0.570 ± 0.023	0.488 ± 0.042	0.707 ± 0.017	0.618 ± 0.01	0.648 ± 0.022	0.663 ± 0.015
XGBoost	0.151 ± 0.018	0.31 ± 0.009	0.3 ± 0.015	0.318 ± 0.015	0.673 ± 0.08	0.606 ± 0.021	0.698 ± 0.009	0.666 ± 0.007	0.681 ± 0.014	0.738 ± 0.007
Random Forest	0.142 ± 0021	0.366 ± 0.01	0.259 ± 0.022	0.298 ± 0.017	0.617 ± 0.011	0.616 ± 0.017	0.769 ± 0.009	0.607 ± 0.014	0.686 ± 0.016	0.695 ± 0.01
**(C)**										
			**F1**		**AU-PRC**		**ACC**		**AU-ROC**	
Linear SVM			0.423 ± 0.011		0.396 ± 0.019		0.423 ± 0.011		0.716 ± 0.001	
Rbf SVM			0.424 ± 0.0		0.322 ± 0.001		0.424 ± 0.0		0.66 ± 0.004	
XGBoost			0.475 ± 0.011		0.495 ± 0.009		0.475 ± 0.011		0.751 ± 0.006	
Random Forest			0.433 ± 0.005		0.47 ± 0.011		0.433 ± 0.005		0.75 ± 0.003	
**(D)**										
	**AU-PRC**					**AU-ROC**				
	**Oncocytoma**	**AML**	**chRCC**	**pRCC**	**ccRCC**	**Oncocytoma**	**AML**	**chRCC**	**pRCC**	**ccRCC**
	**vs. Rest**	**vs. Rest**	**vs. Rest**	**vs. Rest**	**vs. Rest**	**vs. Rest**	**vs. Rest**	**vs. Rest**	**vs. Rest**	**vs. Rest**
Linear SVM	0.141 ± 0.015	0.278 ± 0.022	0.230 ± 0.032	0.288 ± 0.020	0.598 ± 0.04	0.631 ± 0.027	0.734 ± 0.013	0.574 ± 0.042	0.658 ± 0.017	0.673 ± 0.026
Rbf SVM	0.096 ± 0.0	0.148 ± 0.0	0.164 ± 0.0	0.168 ± 0.0	0.424 ± 0.0	0.5 ± 0.0	0.5 ± 0.0	0.5 ± 0.0	0.5 ± 0.0	0.5 ± 0.0
XGBoost	0.124 ± 0.012	0.410 ± 0.029	0.289 ± 0.017	0.294 ± 0.017	0.686 ± 0.012	0.578 ± 0.029	0.748 ± 0.006	0.646 ± 0.013	0.674 ± 0.02	0.750 ± 0.011
Random Forest	0.183 ± 0.033	0.33 ± 0.015	0.258 ± 0.012	0.316 ± 0.017	0.677 ± 0.020	0.645 ± 0.026	0.743 ± 0.014	0.672 ± 0.016	0.672 ± 0.018	0.742 ± 0.013

**Table 3:** The model performance of single-phase CT radiomic feature-based machine learning models in renal cell tumor subtype classification. (**A**) shows the number of CT scans used for training and evaluating machine learning algorithms. (**B**,**C**) show the evaluation performance of machine learning algorithms trained with 10% and 20% of principal components, respectively. We compared the model performance of four difference types of machine learning algorithms in classifying renal cell tumor subtypes with regards to the number of principal components used for training models. We evaluated the model performance of each model in 10-fold cross-validation on a hold-out test set. Sample size indicates the number of labeled images used for training and evaluating machine learning algorithms.

**(A)**																							
				**Arterial Phase**				**Delayed Phase**						**Non-Contrast Phase**					**Portal Phase**				
				**Training Dataset**		**Test Dataset**		**Training Dataset**			**Test Dataset**				**Training Dataset**		**Test Dataset**		**Training Dataset**			**Test Dataset**	
Renal cell tumor subtype				243		75		257			83				280		139		253			188	
oncocytoma (%)				22 (9%)		6 (8%)		23 (9%)			8 (10%)				25 (9%)		13 (10%)		20 (8%)			21 (11%)	
angiomyolipoma (%)				40 (16%)		10 (13%)		42 (16%)			10 (12%)				44 (16%)		20 (14%)		42 (17%)			27 (14%)	
chromophobe renal cell carcinoma (%)				43 (18%)		13 (17%)		42 (16%)			15 (18%)				48 (17%)		25 (18%)		40 (16%)			28 (15%)	
papillary renal cell carcinoma (%)				36 (15%)		14 (19%)		43 (17%)			13 (16%)				48 (17%)		24 (17%)		46 (18%)			37 (20%)	
clear cell renal cell carcinoma (%)				102 (42%)		32 (43%)		107 (42%)			37 (44%)				115 (41%)		57 (41%)		105 (41%)			75 (40%)	
**(B)**																							
	**F1 score**					**AU-PRC**						**ACC**						**AU-ROC**					
**CT Phase**	**Arterial**	**Delayed**	**Non-contrast**		**Portal**	**Arterial**	**Delayed**		**Non-contrast**	**Portal**		**Arterial**	**Delayed**			**Non-contrast**	**Portal**	**Arterial**		**Delayed**	**Non-contrast**		**Portal**
Linear SVM	0.421 ± 0.036	0.378 ± 0.032	0.397 ± 0.02		0.371 ± 0.011	0.42 ± 0.026	0.368 ± 0.032		0.345 ± 0.018	0.341 ± 0.019		0.421 ± 0.036	0.378 ± 0.032			0.397 ± 0.02	0.371 ± 0.011	0.719 ± 0.022		0.676 ± 0.024	0.671 ± 0.019		0.659 ± 0.012
Rbf SVM	0.461 ± 0.028	0.477 ± 0.009	0.425 ± 0.006		0.386 ± 0.004	0.48 ± 0.051	0.416 ± 0.031		0.303 ± 0.024	0.525 ± 0.05		0.461 ± 0.028	0.477 ± 0.09			0.425 ± 0.006	0.386 ± 0.04	0.756 ± 0.027		0.725 ± 0.021	0.67 ± 0.023		0.629 ± 0.018
XGBoost	0.594 ± 0.037	0.479 ± 0.009	0.439 ± 0.015		0.456 ± 0.015	0.608 ± 0.022	0.442 ± 0.18		0.419 ± 0.014	0.463 ± 0.012		0.594 ± 0.037	0.479 ± 0.009			0.439 ± 0.015	0.456 ± 0.015	0.83 ± 0.012		0.728 ± 0.013	0.712 ± 0.006		0.739 ± 0.007
Random Forest	0.478 ± 0.013	0.493 ± 0.015	0.461 ± 0.011		0.441 ± 0.014	0.511 ± 0.016	0.492 ± 0.31		0.471 ± 0.013	0.473 ± 0.015		0.478 ± 0.013	0.493 ± 0.015			0.461 ± 0.011	0.441 ± 0.014	0.778 ± 0.013		0.766 ± 0.016	0.744 ± 0.008		0.756 ± 0.008
**(C)**																							
	**F1 score**					**AU-PRC**						**ACC**						**AU-ROC**					
**CT Phase**	**Arterial**	**Delayed**	**Non-contrast**		**Portal**	**Arterial**	**Delayed**		**Non-contrast**	**Portal**		**Arterial**	**Delayed**			**Non-contrast**	**Portal**	**Arterial**		**Delayed**	**Non-contrast**		**Portal**
Linear SVM	0.396 ± 0.028	0.385 ± 0.027	0.414 ± 0.022		0.297 ± 0.045	0.353 ± 0.048	0.321 ± 0.036		0.354 ± 0.031	0.267 ± 0.031		0.396 ± 0.028	0.385 ± 0.027			0.414 ± 0.022	0.297 ± 0.045	0.669 ± 0.036		0.627 ± 0.032	0.688 ± 0.022		0.581 ± 0.026
Rbf SVM	0.437 ± 0.0	0.462 ± 0.0	0.413 ± 0.0		0.382 ± 0.0	0.337 ± 0.01	0.337 ± 0.016		0.317 ± 0.002	0.296 ± 0.007		0.437 ± 0.0	0.462 ± 0.0			0.413 ± 0.0	0.382 ± 0.0	0.677 ± 0.006		0.67 ± 0.008	0.665 ± 0.005		0.633 ± 0.007
XGBoost	0.531 ± 0.02	0.466 ± 0.029	0.354 ± 0.024		0.447 ± 0.019	0.565 ± 0.014	0.507 ± 0.021		0.382 ± 0.022	0.429 ± 0.013		0.531 ± 0.002	0.466 ± 0.029			0.354 ± 0.024	0.447 ± 0.019	0793 ± 0.005		0.766 ± 0.001	0.686 ± 0.023		0.732 ± 0.008
Random Forest	0.504 ± 0.022	0.47 ± 0.016	0.45 ± 0.006		0.427 ± 0.013	0.52 ± 0.026	0.529 ± 0.02		0.433 ± 0.009	0.449 ± 0.014		0.504 ± 0.022	0.497 ± 0.016			0.45 ± 0.006	0.427 ± 0.013	0.797 ± 0.018		0.73 ± 0.014	0.16 ± 0.007		0.735 ± 0.007

**Table 4:** The model performance of single-phase CT radiomics feature-based machine learning models in renal cell tumor subtype classification (**A**,**B**) show the AU-PRC and AU-ROC, respectively, of singe-phase CT radiomics feature-based machine learning models trained with 10% of principal components in classifying a specific subtype against other subtypes. (**C**,**D**) show the AU-PRC and AU-ROC, respectively, of models trained with 20% of principal components in the same task.

**(A)**																				
	**Arterial**					**Delayed**					**Non-Contrast**					**Portal**				
**CT Phase**	**Onco-** **cytoma**	**AML**	**chRCC**	**pRCC**	**ccRCC**	**Onco-** **cytoma**	**AML**	**chRCC**	**pRCC**	**ccRCC**	**Onco-** **cytoma**	**AML**	**chRCC**	**pRCC**	**ccRCC**	**Onco-** **cytoma**	**AML**	**chRCC**	**pRCC**	**ccRCC**
Linear SVM	0.147 ± 0.068	0.290 ± 0.077	0.280 ± 0.028	0.298 ± 0.078	0.656 ± 0.065	0.151 ± 0.056	0.205 ± 0.041	0.310 ± 0.079	0.260 ± 0.059	0.564 ± 0.037	0.109 ± 0.016	0.214 ± 0.025	0.202 ± 0.051	0.206 ± 0.036	0.503 ± 0.030	0.141 ± 0.016	0.194 ± 0.027	0.217 ± 0.019	0.275 ± 0.028	0.550 ± 0.054
Rbf SVM	0.133 ± 0.038	0.382 ± 0.135	0.285 ± 0.041	0.598 ± 0.119	0.638 ± 0.085	0.128 ± 0.059	0.241 ± 0.044	0.268 ± 0.1	0.421 ± 0.095	0.547 ± 0.07	0.084 ± 0.008	0.340 ± 0.105	0.170 ± 0.013	0.168 ± 0.038	0.463 ± 0.034	0.117 ± 0.014	0.190 ± 0.060	0.148 ± 0.008	0.200 ± 0.025	0.384 ± 0.048
XGBoost	0.089 ± 0.017	0.605 ± 0.076	0.590 ± 0.063	0.393 ± 0.1	0.757 ± 0.033	0.105 ± 0.01	0.420 ± 0.028	0.351 ± 0.038	0.346 ± 0.049	0.586 ± 0.023	0.111 ± 0.015	0.401 ± 0.022	0.320 ± 0.033	0.196 ± 0.016	0.556 ± 0.024	0.189 ± 0.033	0.411 ± 0.016	0.273 ± 0.035	0.333 ± 0.31	0.682 ± 0.02
Random Forest	0.163 ± 0.026	0.384 ± 0.035	0.265 ± 0.049	0.413 ± 0.052	0.711 ± 0.026	0.158 ± 0.023	0.356 ± 0.021	0.277 ± 0.038	0.344 ± 0.093	0.712 ± 0.046	0.149 ± 0.029	0.499 ± 0.023	0.239 ± 0.027	0.238 ± 0.019	0.637 ± 0.039	0.214 ± 0.032	0.479 ± 0.052	0.271 ± 0.027	0.295 ± 0.016	0.643 ± 0.02
**(B)**																				
	**Arterial**					**Delayed**					**Non-Contrast**					**Portal**				
**CT Phase**	**Onco-** **cytoma**	**AML**	**chRCC**	**pRCC**	**ccRCC**	**Onco-** **cytoma**	**AML**	**chRCC**	**pRCC**	**ccRCC**	**Onco-** **cytoma**	**AML**	**chRCC**	**pRCC**	**ccRCC**	**Onco-** **cytoma**	**AML**	**chRCC**	**pRCC**	**ccRCC**
Linear SVM	0.625 ± 0.149	0.681 ± 0.073	0.572 ± 0.053	0.687 ± 0.056	0.663 ± 0.049	0.584 ± 0.094	0.631 ± 0.048	0.561 ± 0.084	0.645 ± 0.067	0.595 ± 0.042	0.529 ± 0.056	0.685 ± 0.049	0.465 ± 0.075	0.492 ± 0.049	0.595 ± 0.022	0.561 ± 0.040	0.639 ± 0.047	0.537 ± 0.053	0.584 ± 0.046	0.655 ± 0.044
Rbf SVM	0.133 ± 0.038	0.382 ± 0.135	0.285 ± 0.041	0.598 ± 0.119	0.638 ± 0.084	0.444 ± 0.100	0.570 ± 0.079	0.547 ± 0.117	0.699 ± 0.113	0.635 ± 0.082	0.458 ± 0.064	0.580 ± 0.139	0.415 ± 0.065	0.452 ± 0.090	0.524 ± 0.047	0.504 ± 0.055	0.424 ± 0.133	0.459 ± 0.018	0.442 ± 0.018	0.500 ± 0.074
XGBoost	0.507 ± 0.05	0.843 ± 0.028	0.775 ± 0.032	0.781 ± 0.04	0.824 ± 0.023	0.500 ± 0.042	0.715 ± 0.019	0.707 ± 0.029	0.616 ± 0.041	0.675 ± 0.011	0.513 ± 0.049	0.693 ± 0.02	0.668 ± 0.026	0.522 ± 0.014	0.645 ± 0.018	0.580 ± 0.033	0.785 ± 0.016	0.659 ± 0.024	0.667 ± 0.015	0.761 ± 0.013
Random Forest	0.664 ± 0.052	0.755 ± 0.045	0.636 ± 0.048	0.769 ± 0.033	0.775 ± 0.026	0.644 ± 0.059	0.749 ± 0.025	0.659 ± 0.037	0.637 ± 0.113	0.785 ± 0.037	0.603 ± 0.056	0.780 ± 0.026	0.585 ± 0.019	0.577 ± 0.038	0.706 ± 0.025	0.669 ± 0.033	0.806 ± 0.017	0.651 ± 0.022	0.656 ± 0.023	0.731 ± 0.017
**(C)**																				
	**Arterial**					**Delayed**					**Non-Contrast**					**Portal**				
**CT Phase**	**Onco-** **cytoma**	**AML**	**chRCC**	**pRCC**	**ccRCC**	**Onco-** **cytoma**	**AML**	**chRCC**	**pRCC**	**ccRCC**	**Onco-** **cytoma**	**AML**	**chRCC**	**pRCC**	**ccRCC**	**Onco-** **cytoma**	**AML**	**chRCC**	**pRCC**	**ccRCC**
Linear SVM	0.106 ± 0.06	0.217 ± 0.085	0.188 ± 0.075	0.207 ± 0.063	0.583 ± 0.059	0.111 ± 0.025	0.189 ± 0.082	0.196 ± 0.049	0.218 ± 0.054	0.469 ± 0.061	0.129 ± 0.030	0.331 ± 0.055	0.207 ± 0.04	0.199 ± 0.035	0.512 ± 0.042	0.111 ± 0.017	0.163 ± 0.029	0.17 ± 0.018	0.217 ± 0.025	0.408 ± 0.04
Rbf SVM	0.185 ± 0.072	0.215 ± 0.046	0.165 ± 0.007	0.168 ± 0.015	0.420 ± 0.019	0.18 ± 0.049	0.212 ± 0.097	0.162 ± 0.024	0.181 ± 0.043	0.412 ± 0.031	0.091 ± 0.0	0.147 ± 0.0	0.175 ± 0.0	0.175 ± 0.0	0.413 ± 0.0	0.111 ± 0.009	0.248 ± 0.034	0.154 ± 0.009	0.205 ± 0.024	0.361 ± 0.02
XGBoost	0.177 ± 0.056	0.584 ± 0.037	0.261 ± 0.023	0.393 ± 0.067	0.726 ± 0.026	0.184 ± 0.077	0.399 ± 0.048	0.239 ± 0.026	0.34 ± 0.036	0.714 ± 0.032	0.119 ± 0.025	0.443 ± 0.048	0.22 ± 0.023	0.222 ± 0.025	0.549 ± 0.029	0.147 ± 0.016	0.452 ± 0.038	0.318 ± 0.045	0.394 ± 0.027	0.552 ± 0.025
Random Forest	0.249 ± 0.072	0.451 ± 0.028	0.326 ± 0.079	0.368 ± 0.076	0.671 ± 0.041	0.136 ± 0.02	0.533 ± 0.072	0.252 ± 0.052	0.388 ± 0.051	0.741 ± 0.032	0.141 ± 0.025	0.395 ± 0.021	0.212 ± 0.04	0.221 ± 0.032	0.627 ± 0.022	0.123 ± 0.021	0.428 ± 0.044	0.261 ± 0.027	0.318 ± 0.031	0.622 ± 0.034
**(D)**																				
	**Arterial**					**Delayed**					**Non-Contrast**					**Portal**				
**CT Phase**	**Onco-** **cytoma**	**AML**	**chRCC**	**pRCC**	**ccRCC**	**Onco-** **cytoma**	**AML**	**chRCC**	**pRCC**	**ccRCC**	**Onco-** **cytoma**	**AML**	**chRCC**	**pRCC**	**ccRCC**	**Onco-** **cytoma**	**AML**	**chRCC**	**pRCC**	**ccRCC**
Linear SVM	0.509 ± 0.135	0.566 ± 0.135	0.478 ± 0.132	0.537 ± 0.139	0.580 ± 0.061	0.466 ± 0.094	0.514 ± 0.161	0.481 ± 0.079	0.475 ± 0.094	0.5 ± 0.066	0.598 ± 0.068	0.744 ± 0.036	0.53 ± 0.044	0.488 ± 0.062	0.605 ± 0.034	0.46 ± 0.058	0.495 ± 0.079	0.484 ± 0.043	0.493 ± 0.041	0.491 ± 0.055
Rbf SVM	0.497 ± 0.085	0.56 ± 0.105	0.457 ± 0.045	0.441 ± 0.039	0.43 ± 0.049	0.492 ± 0.061	0.529 ± 0.115	0.429 ± 0.066	0.433 ± 0.082	0.387 ± 0.072	0.5 ± 0.0	0.5 ± 0.	0.5 ± 0.	0.5 ± 0.	0.5 ± 0.	0.483 ± 0.041	0.587 ± 0.042	0.412 ± 0.05	0.518 ± 0.031	0.409 ± 0.045
XGBoost	0.489 ± 0.039	0.805 ± 0.028	0.671 ± 0.027	0.707 ± 0.029	0.751 ± 0.015	0.594 ± 0.054	0.762 ± 0.019	0.630 ± 0.016	0.766 ± 0.029	0.731 ± 0.023	0.503 ± 0.045	0.747 ± 0.021	0.545 ± 0.022	0.607 ± 0.041	0.632 ± 0.025	0.560 ± 0.028	0.796 ± 0.019	0.678 ± 0.031	0.726 ± 0.021	0.648 ± 0.031
Random Forest	0.648 ± 0.074	0.78 ± 0.028	0.717 ± 0.077	0.717 ± 0.077	0.716 ± 0.024	0.541 ± 0.062	0.799 ± 0.033	0.609 ± 0.045	0.712 ± 0.061	0.777 ± 0.03	0.581 ± 0.037	0.757 ± 0.016	0.513 ± 0031	0.554 ± 0.048	0.723 ± 0.028	0.567 ± 0.049	0.804 ± 0.015	0.658 ± 0.036	0.669 ± 0.025	0.706 ± 0.019

## Data Availability

The relevant code is freely available for reproducibility (https://github.com/Transconnectome/Kidney_Radiomics, accessed on 16 October 2023).

## Supplementary Materials

The following supporting information can be downloaded at https://www.mdpi.com/article/10.3390/diagnostics15111365/s1, Figure S1: Scree plots of principal components derived from PCA using 10% of selected radiomics features via F-test for each CT phase, Figure S2: Scree plots of principal components derived from PCA using 10% of selected radiomics features via F-test for each CT phase, Figure S3: Renal cell tumor subtype classification performance of XGBoost trained with 10% of principal components derived from arterial phase CT scan radiomic features, Figure S4: Renal cell tumor subtype classification performance of XGBoost trained with 10% of principal components derived from delayed phase CT scan radiomic features, Figure S5: Renal cell tumor subtype classification performance of XGBoost trained with 10% of principal components derived from non-contrast phase CT scan radiomic features, Figure S6: Renal cell tumor subtype classification performance of XGBoost trained with 10% of principal components derived from portal phase CT scan radiomic features
