# Supplementary material for: Computed Tomography-Based Radiomics Diagnostic Model for Fat-Poor Small Renal Tumor Subtypes
Source: Diagnostics (Basel). 2025 May 28;15(11):1365. doi: 10.3390/diagnostics15111365 (PMC12155376; doi:10.3390/diagnostics15111365)
Supplement: Supplementary file 1 [file diagnostics-15-01365-s001.zip › diagnostics-3495949-supplementary.pdf]

Supplementary Materials

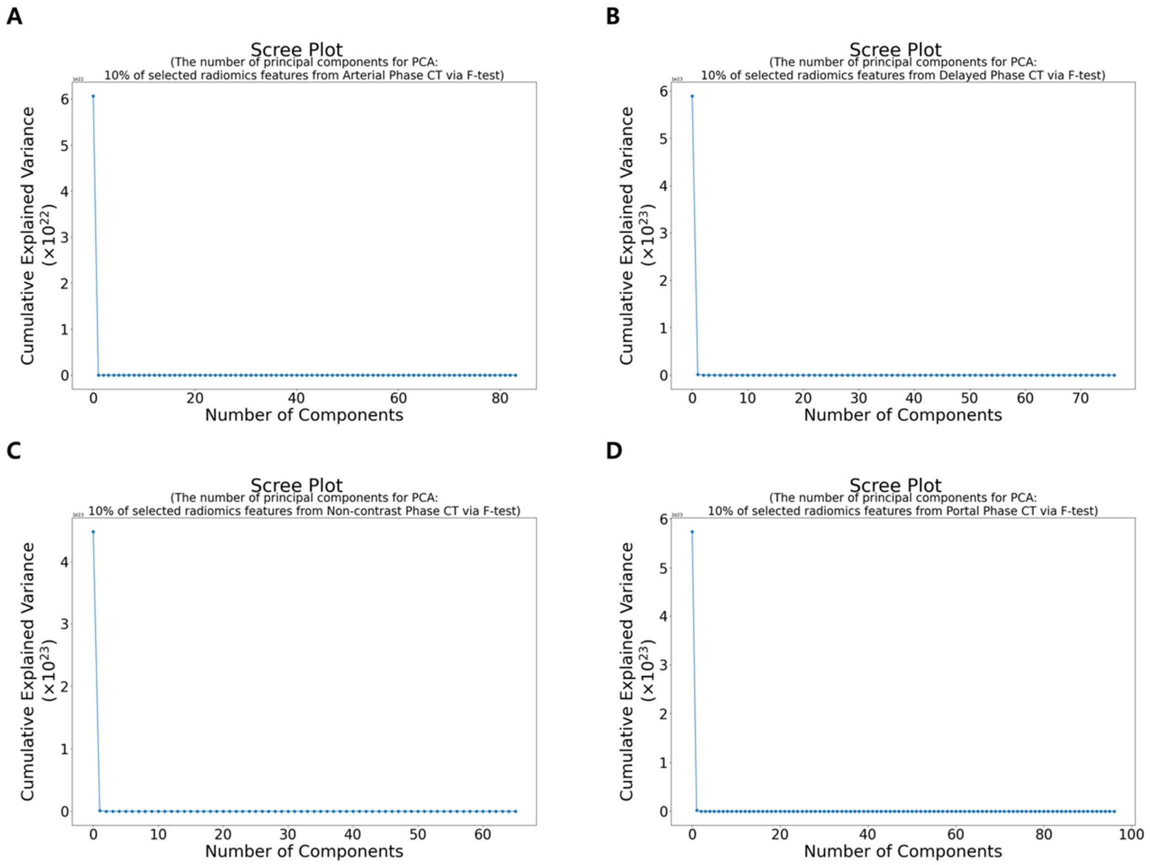

Supplementary Figure S1. Scree plots of principal components derived from PCA using 10% of selected radiomics features via F-test for each CT phase: (A) Arterial, (B) Delayed, (C) Non-contrast, and (D) Portal phase scans.

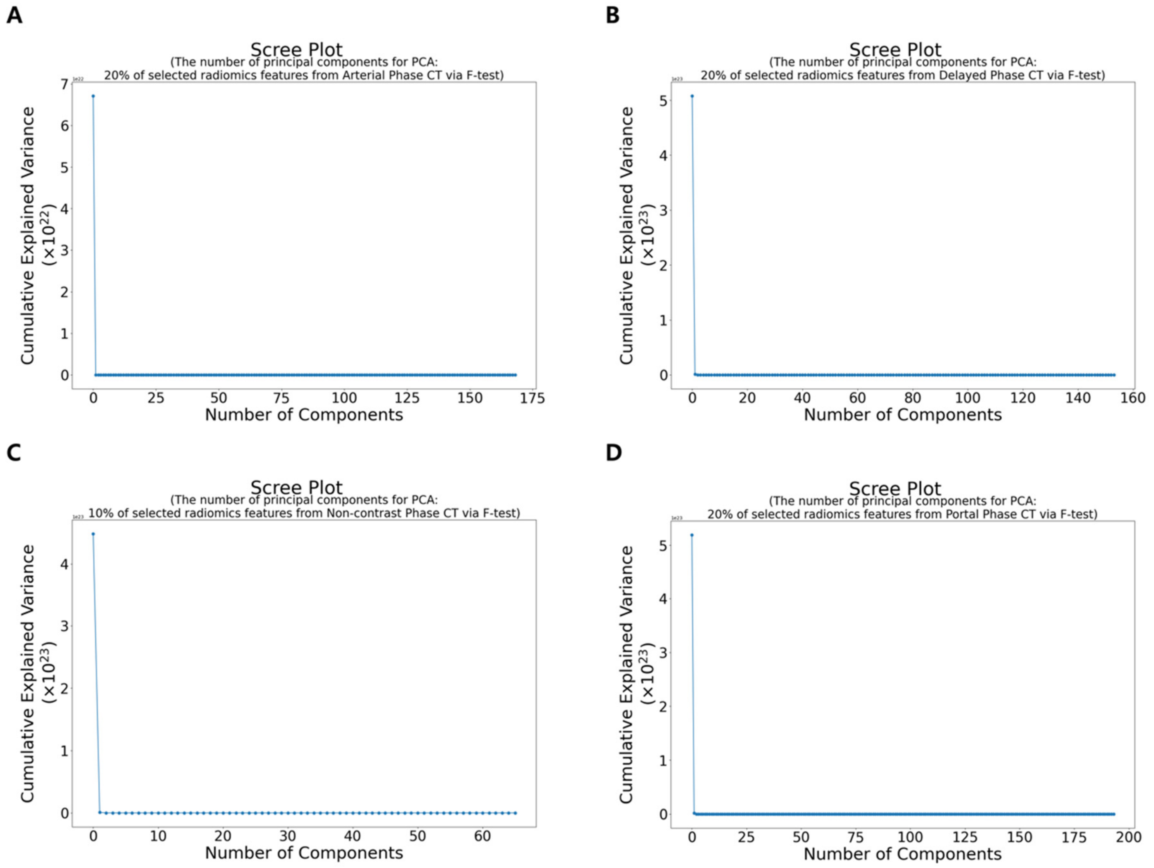

Supplementary Figure S2. Scree plots of principal components derived from PCA using 20% of selected radiomics features via F-test for each CT phase: (A) Arterial, (B) Delayed, (C) Non-contrast, and (D) Portal phase scans.

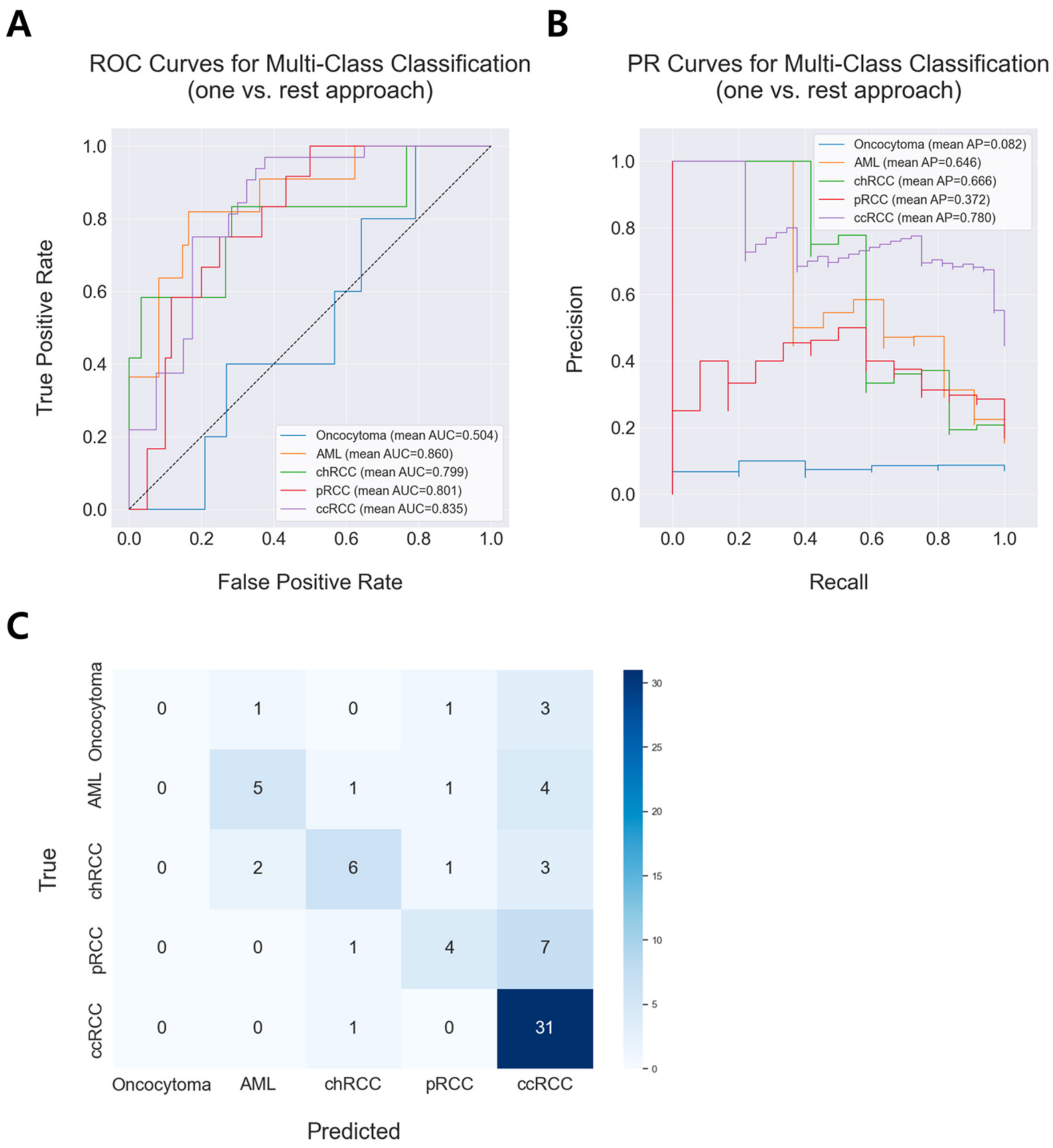

Supplementary Figure S3. Renal cell tumor subtype classification performance of XGBoost trained with 10% of principal components derived from arterial phase CT scan radiomic features

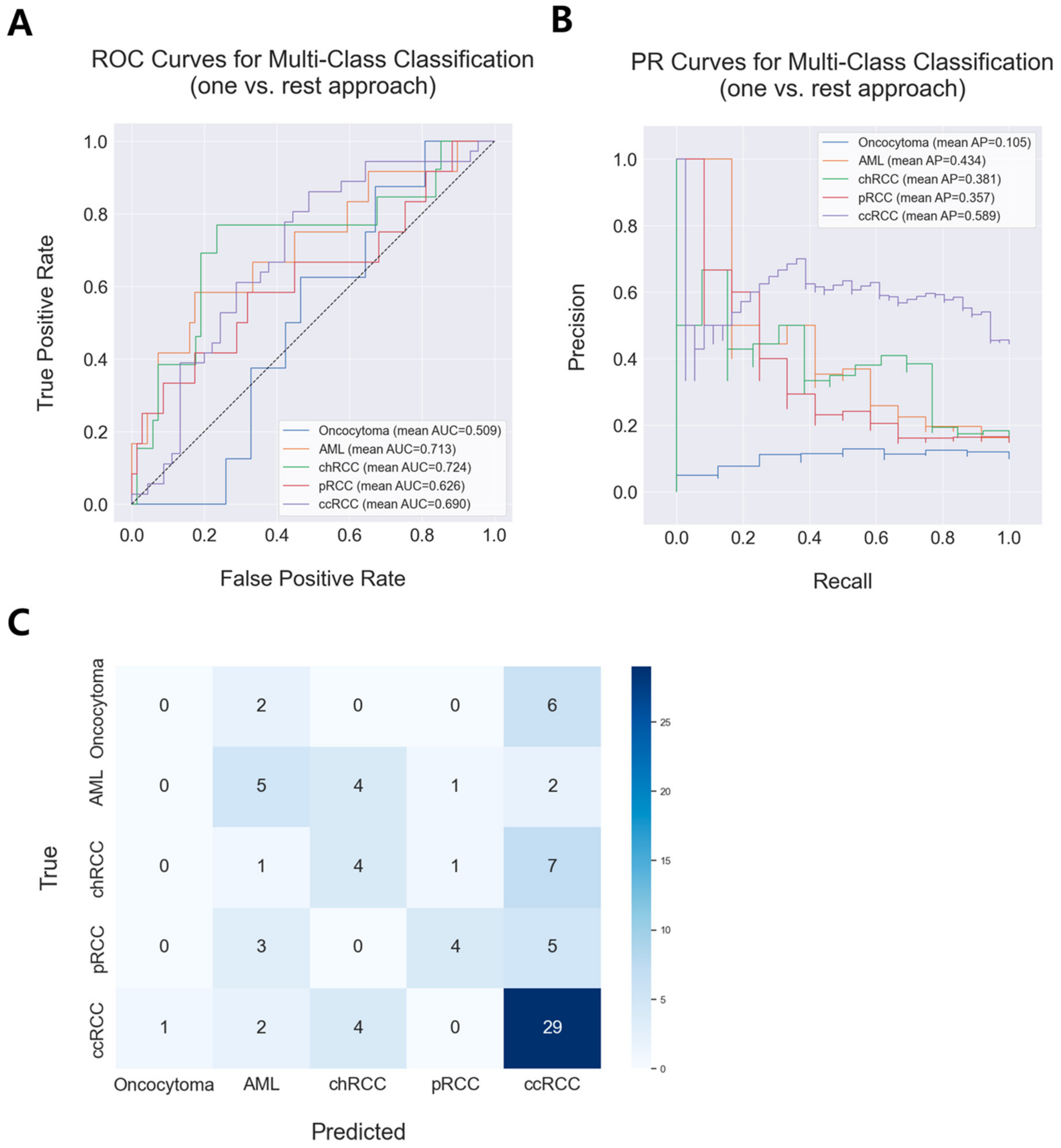

Supplementary Figure S4. Renal cell tumor subtype classification performance of XGBoost trained with 10% of principal components derived from delayed phase CT scan radiomic features

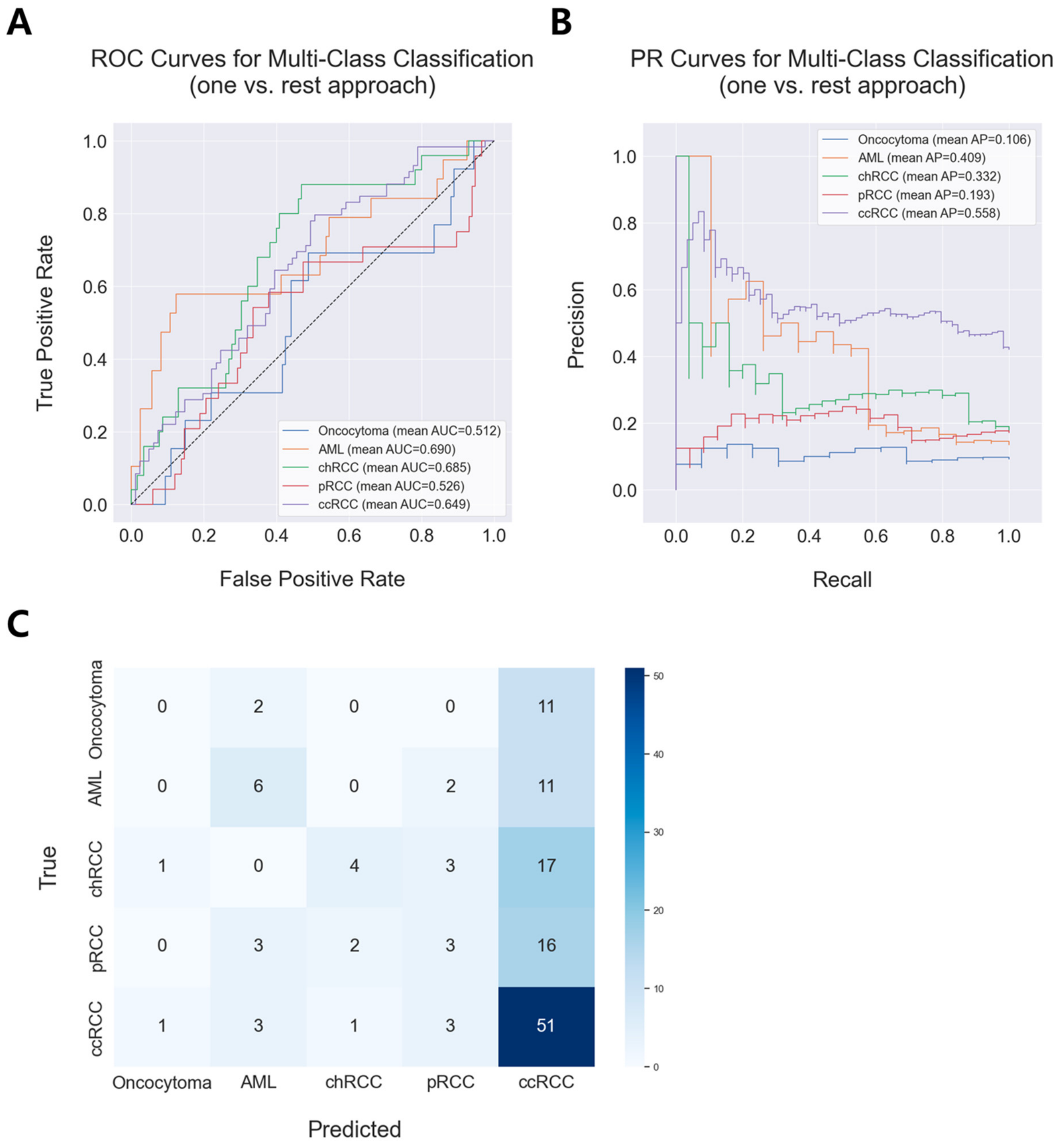

Supplementary Figure S5. Renal cell tumor subtype classification performance of XGBoost trained with 10% of principal components derived from non-contrast phase CT scan radiomic features

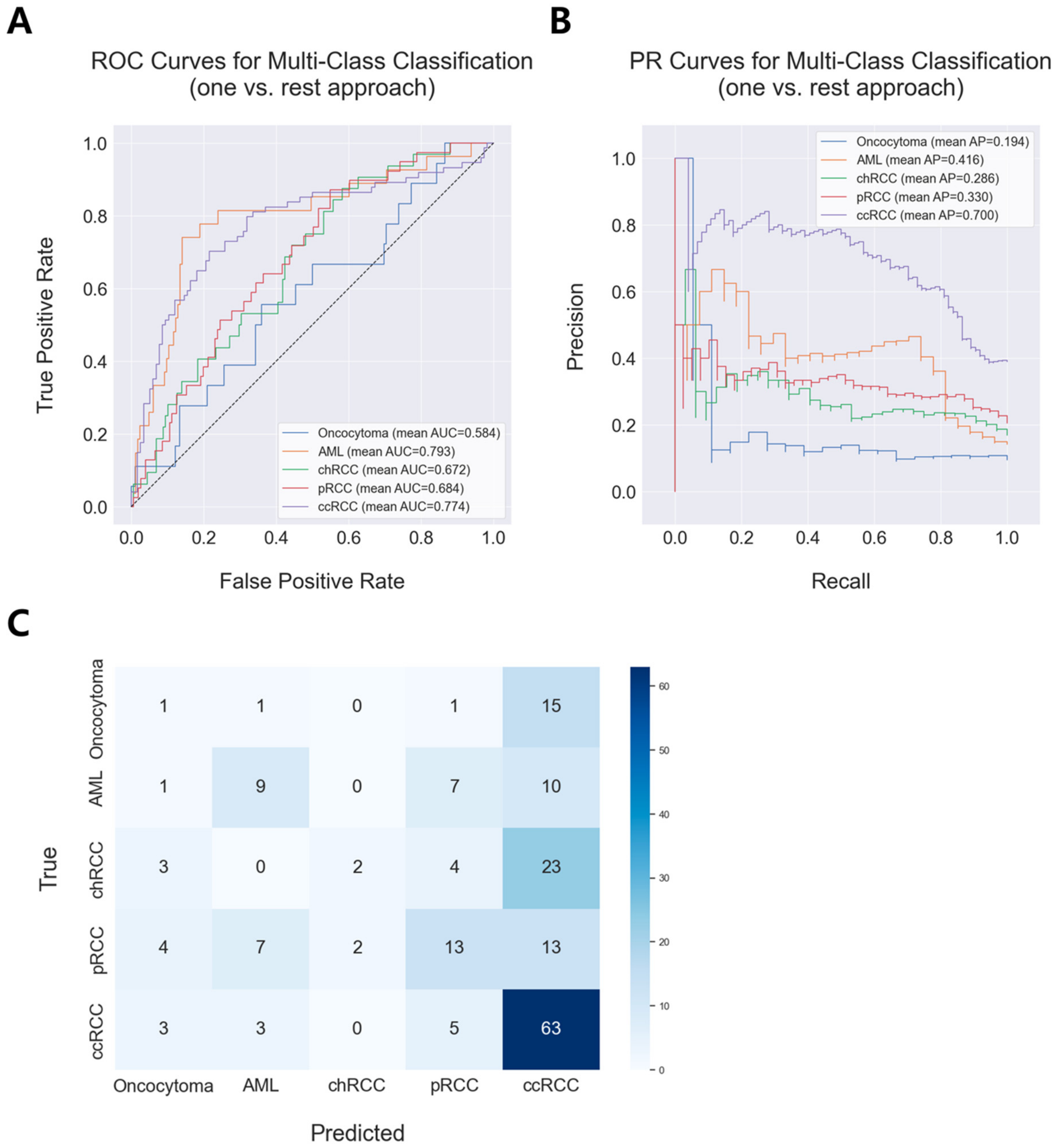

Supplementary Figure S6. Renal cell tumor subtype classification performance of XGBoost trained with 10% of principal components derived from portal phase CT scan radiomic features
